# SARS-CoV-2 infection among educational staff in Berlin, Germany, June to December 2020

**DOI:** 10.2807/1560-7917.ES.2022.27.11.2100524

**Published:** 2022-03-17

**Authors:** Sophia Kindzierski, Welmoed van Loon, Stefanie Theuring, Franziska Hommes, Eberhard Thombansen, Malik Böttcher, Harald Matthes, Heike Rössig, David Weiger, Christof Wiesmann, Tobias Kurth, Valerie Kirchberger, Joachim Seybold, Frank P Mockenhaupt, Maximilian Gertler

**Affiliations:** 1 Medical Directorate, Charité – Universitätsmedizin Berlin, corporate member of Freie Universität Berlin and Humboldt-Universität zu Berlin, Berlin, Germany; 2 Institute of Tropical Medicine and International Health, Charité – Universitätsmedizin Berlin, corporate member of Freie Universität Berlin and Humboldt-Universität zu Berlin, Berlin, Germany; 3 Vivantes Hospital Group, Berlin, Germany; 4 Gemeinschaftskrankenhaus Havelhöhe, Berlin, Germany; 5 Institute of Public Health, Charité – Universitätsmedizin Berlin, corporate member of Freie Universität Berlin and Humboldt-Universität zu Berlin, Berlin, Germany; * These authors contributed equally to this manuscript and share first authorship.

**Keywords:** SARS-CoV-2, day care, school, teacher, Germany, screening

## Abstract

**Background:**

SARS-CoV-2 infections in preschool and school settings potentially bear occupational risks to educational staff.

**Aim:**

We aimed to assess the prevalence of SARS-CoV-2 infection in teachers and preschool educators and at identifying factors associated with infection.

**Methods:**

We analysed cross-sectional data derived from 17,448 voluntary, PCR-based screening tests of asymptomatic educational staff in Berlin, Germany, between June and December 2020 using descriptive statistics and a logistic regression model.

**Results:**

Participants were largely female (73.0%), and median age was 41 years (range: 18-78). Overall, SARS-CoV-2 infection proportion was 1.2% (95% CI: 1.0–1.4). Proportion of positive tests in educational staff largely followed community incidence until the start of the second pandemic wave, when an unsteady plateau was reached. Then, the proportion of positive tests in a (concurrent) population survey was 0.9% (95% CI: 0.6–1.4), 1.2% (95% CI: 0.8–1.8) in teachers and 2.6% (95% CI: 1.6–4.0) in preschool educators. Compared with teachers, increased odds of infection were conferred by being a preschool educator (adjusted odds ratio (aOR): 1.6; 95% CI: 1.3–2.0) and by contact with a SARS-CoV-2 infected individual outside of work (aOR: 3.0; 95% CI: 1.5–5.5). In a step-wise backward selection, the best set of associated factors with SARS-CoV-2 infection involved age, occupation, and calendar week.

**Conclusions:**

These results indicate that preschool educators bear increased odds of SARS-CoV-2 infection compared with teachers. At the same time, the private environment appeared to be a relevant source of SARS-CoV-2 infection for educational staff in 2020.

## Introduction

The operation of schools and preschool centres during the coronavirus disease (COVID-19) pandemic is subject to intense debate. Teachers and preschool educators are front-line workers in the education system. Considerations of the children’s contagiousness and potential transmission chains have led to public health concerns about safe school attendance and workplace security. Such concerns, in turn, impact the functionality of schooling and childcare [1]. While the influence of opened or closed educational facilities over the course of the pandemic has been investigated by modelling [2,3] and observational studies [4-7], with partially contradicting findings, the actual occupational risk of educational staff is less clear. Moreover, repeatedly changing infection prevention and control (IPC) measures, such as mask-wearing obligations or dividing classes, also make it difficult to draw firm conclusions.

The present study aimed at assessing the proportion of positive tests for severe acute respiratory syndrome coronavirus 2 (SARS-CoV-2) infections in school teachers and preschool educators using a screening offer for asymptomatic educational staff in the period between June and December 2020 at five testing sites throughout Berlin. Moreover, we evaluated the association between occupation (teachers vs preschool educators) and recent contact to an individual with a confirmed SARS-CoV-2 infection in order to identify the combination of variables in the dataset that best described SARS-CoV-2 infection status.

## Methods

### Study setting and participants

The Senate of Berlin initiated a free voluntary screening programme for asymptomatic educational staff, which started on 8 June 2020 and was discontinued on 31 December 2020. Testing was available at five specialised testing sites in Berlin: Site A. Charité – Universitätsmedizin Berlin (8 June–13 December); Site B. Vivantes Wenckebach-Klinikum (6 July–30 December); Site C. Vivantes Klinikum Spandau (6 July–30 December); Site D. Vivantes Prenzlauer Berg (6 July–30 December) and Site E. Gemeinschaftskrankenhaus Havelhoehe (28 July–30 December). Educational staff in Berlin, which included permanent staff, trainees and volunteers, were invited to participate. The respective invitations and detailed information were sent to all schools and preschools (childcare for children up to 5 years of age) and forwarded to the facilities’ staff. The testing was offered 5 days per week, from Mondays to Fridays, even during school vacation periods.

### Epidemiological situation and public health measures

From June–August 2020, the weekly incidence of COVID-19 cases [8] in Berlin was low, i.e. around 3–13 per 100,000 inhabitants. Starting mid-September, incidence sharply increased and peaked between mid-November and mid-December 2020, with weekly incidence figures of up to 227 per 100,000 inhabitants (week 47). With increasing community incidence, progressive IPC measures became mandatory for the population, such as facemask obligations or access limitations in certain public locations. By November 2020, a national ‘lockdown light’ was implemented, comprising closure of gastronomic and cultural places as well as restrictions for social meetings and travelling.

During the study period, schools and preschools remained continuously open for symptomless children, but closed during regular school vacation periods. School operations, which had basic IPC hygiene measures in place since reopening in May 2020, were also influenced by increasing community incidence leading to e.g. expansion of facemask wearing from way-to-class to use-in-class. As at the end of October 2020, the following measures were decreed: obligatory facemask use in and outside of the classroom, classes split into smaller groups, and open-window ventilation. In preschools, IPC measures were less strict and included mainly regulation of intra-staff and parental contacts, but with no obligatory mask wearing for children or preschool educators. A full lockdown was imposed on 16 December 2020, closing schools, preschools and cultural institutions, although offices and other places of work remained open.

### Study design

The two-part screening consisted of an RT-PCR-based SARS-CoV-2 test and questionnaire regarding the participants’ medical situation and occupational context. Data were analysed separately for teachers (those working with children and adolescents aged 6–18 years) and preschool educators (those working with children aged 0–5 years). Individuals who were mildly symptomatic at presentation or were primary contacts of SARS-CoV-2-positive individuals were not rejected from our study. Repeat tests were allowed at a monthly interval.

### Data and sample collection

Basic demographic data, e.g. age, sex, occupation, and PCR test results were collected. Because of a decentralised implementation approach of the screening, only two testing sites (A and E) collected further data. At Site A, a web app-based questionnaire collected information on symptoms, previous contacts and IPC measures (Supplementary Table S3: Questionnaire for the screening of special occupational/population groups). At Site E, a paper-based questionnaire collected information on symptoms and previous contacts.

From each participant, a nasopharyngeal, oropharyngeal or combined swab was taken by a healthcare professional; SARS-CoV-2 infection was assessed. The samples from Sites A-D were analysed by RT-PCR using the cobas 6800/8800 system (Roche Diagnostics, Mannheim, Germany), and the samples from site E, by RT-PCR using AltoStar® SARS-CoV-2 RT-PCR Kit 1.5 RUO (Altona Diagnostics, Hamburg, Germany). According to German infection prevention laws, all positive test results must be reported to the local health authority.

### Data analysis

Participants who identified as working in educational settings, but not as teachers or preschool educators, and participants reporting an age of less than 18 and more than 79 years were excluded from the analysis. Basic characteristics were described by median and range, proportion and 95% confidence intervals (CI), and univariate odds ratio (OR) and 95% (CI), as applicable. For proportions, denominators represent the number of unique individuals with available data. We estimated the association of occupation and recent contact to a SARS-CoV-2 infected individual using a binomial logistic regression model adjusting for the potential confounding variables sex, age of the teacher or preschool educator and calendar week as covariates and random intercept for the five testing sites. We further evaluated the association of contact history in the private context and confirmed SARS-CoV-2 status also adjusting for ‘contact history at work’. We explored the combination of variables in the dataset that best described SARS-CoV-2 infection status with a backward stepwise selection based on the Akaike information criterion. The likelihood of variable selection was further evaluated by repeating the variable selection using the bootstrap technique (Supplementary Analysis S1: Data collected at Site A and E). All analyses were done in R version 3.6.3 (R Foundation, Vienna, Austria).

### Ethical statement

This study was performed in line with the principles of the Declaration of Helsinki. The study was approved by the Charité - Universitätsmedizin Berlin Ethics Committee (EA1/313/20). All participants provided written consent.

## Results

### Study population

Between 8 June 2020 and 31 December 2020, 18,941 tests for SARS-CoV-2 were performed (Figure). A total of 17,448 participants were included for analysis; 74.6% were teachers (n = 13,012) and 25.4% were preschool educators (n = 4,436). The number of tests per site were as follows: Site A had 2,287 tests (13.1%), Site B had 4,542 tests (26.0%), Site C had 2,908 tests (16.7%), Site D had 4,427 tests (25.4%) and Site E had 3,284 tests (18.8%). Repeat testing was recorded at Site A, and 14.8% (286/1,936) of participants were tested more than once. Symptom data was collected at Sites A and E, where 5.4% (301/5,539) of participants reported to have at least one symptom compatible with a SARS-CoV-2 infection. 

**Figure:**
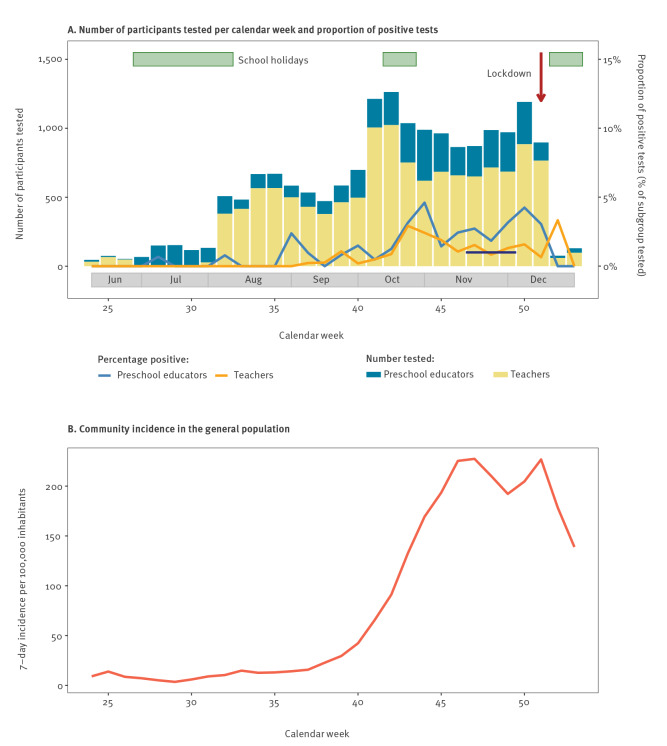
Proportion of positive tests of SARS-CoV-2 infection among educational staff and community incidence in Berlin, Germany, June–December 2020 (n = 17,448)

SARS-CoV-2: severe acute respiratory syndrome coronavirus 2.

Panel A shows the number of participants tested for SARS-CoV-2 per calendar week as well as the proportion of educational staff who tested positive. School holidays are indicated by green bars. The red arrow indicates the ‘lockdown light’ in Berlin, which took effect on 2 November 2020. The black line indicates the proportion of SARS-Cov-2 infected individuals in a population survey in central Berlin during the respective sampling period [10]. Panel B shows the community incidence in Berlin as the 7-day incidence per 100,000 inhabitants. Data was provided by the Senate of Health and Social Affairs, Berlin (LAGeSo) [8].

Participants were primarily women (73.0% (12,686/17,386)), and the median age was 41 years (range: 18–78, including trainees and volunteers). More preschool educators than teachers were women (82.4% (3,648/4,428) vs 69.7% (9,038/12,958), and they were younger (median age: 40 years (range: 18–73)) than teachers (41 years; range: 18–78)). Sex, age, and occupation differed only slightly between testing sites (Supplementary Table S1: Distribution of test population at each test site). Sex and age reflect the general distribution of Berlin teachers and preschool educators, except for teachers´ age, which was slightly lower in our cohort than the average age of teachers in Berlin (42.9 vs 46.7 years) [9].

### Proportion of positive SARS-CoV-2 tests

During the study period, 1.2% (95% CI: 1.0–1.4; 210/17,448) of tests were positive for SARS-CoV-2, relatively evenly distributed across testing sites (range: 1.0–1.7%). At the two sites documenting symptoms (A and E), 16.7% (11/66) of educational staff with a positive test reported symptoms as compared with 5.3% (290/5,473) in those who tested negative.

We examined the proportion of positive SARS-CoV-2 tests in educational staff (Figure). The proportion of positive tests in teachers (overall 1.0%; 95% CI: 0.8–1.1) was zero until mid-September (week 37), when it started to increase to reach a preliminary peak (2.9%; 95% CI: 1.8–4.4) by mid-October (week 43). In the smaller group of preschool educators, proportion of positive tests (overall 1.9%; 95% CI: 1.5–2.4) exceeded zero at several points until mid-September, then also increased and peaked end of October (4.6%; 95% CI: 2.7–7.3). These increases in proportion largely paralleled the community incidence. Yet, in both teachers and preschool educators, positive test proportions dropped 3–4 weeks before the highest community incidence, reached an unsteady 4-week plateau with another peak at the time the lockdown was implemented. During the plateau-phase (weeks 47–49), the proportion of SARS-CoV-2 infected individuals in a representative, unweighted adult population survey in the central city district of Berlin was 0.9% (95% CI: 0.6–1.4; 21/2,287) [10]. The simultaneous positive test proportion in teachers was similar at 1.2% (95% CI: 0.8–1.8; 24/1,933) and was 2.6% among preschool educators (95% CI: 1.6–4.0; 19/739).

### Factors associated with SARS-CoV-2 infection

We observed increased odds for preschool educators, as well as for all educational staff with reported non-work-related contacts to an individual with confirmed SARS-CoV-2 infection and for younger staff (Table). The latter was true for both preschool educators (contacts outside of work, OR: 4.2 (95% CI: 1.1–13.3); increasing age, OR: 0.97 per additional year (95% CI: 0.95–0.99)) and teachers (contacts outside of work, OR: 10.6 (95% CI: 1.7–46.5); increasing age, OR: 0.97 per additional year (95% CI: 0.95–0.98)). When estimating the association of occupation (teacher vs preschool educator, adjusted for age, sex, calendar week, and testing site as random effect) and contact history (same adjustment set, with contact history in the private and work context both added to the same model), we found that reported contacts to an individual with a confirmed SARS-CoV-2 infection at work was not associated with SARS-CoV-2 infection (adjusted OR (aOR): 0.9; 95% CI: 0.4–1.8), but contacts beyond the workplace tripled the odds (aOR: 3.0; 95% CI: 1.5–5.5). Being a preschool educator increased the odds as well (aOR: 1.6; 95% CI: 1.3–2.0). Similar results were observed after re-running the analysis after excluding facilitating staff, i.e. not being a teacher or educator (data only collected at testing site A and E) (Supplementary Table S2: Characteristics of excluded participants and included participants from testing site A and E and Figure S1: Comparison of effect estimates for SARS-CoV-2 infection in a dataset with and without exclusion of non-teachers and non-preschool educators). We did not find any association of IPC measure adherence with SARS-CoV-2 infection in the study subset with respectively available data (data not shown). For the subset of individuals with available workplace data (Site A, n = 2,045), proportion of positive tests was 1.7% (95% CI: 1.0–2.6; 19/1,124), 1.4% (95% CI: 0.5–2.9; 6/440), and 0.8% (95% CI: 0.2–2.1; 4/480) in preschool educators, primary school teachers and secondary school teachers, respectively.

**Table:** Factors associated with SARS-CoV-2 infection among educational staff in Berlin, Germany, June to December 2020 (n = 17,448)

Characteristics	Result of SARS-CoV-2 PCR				OR	95% CI
	Positive (n = 210)		Negative (n = 17,238)			
	n	%	n	%		
Sex^a^						
Male	58	1.2	4,642	98.8	Ref.	NA
Female	151	1.2	12,535	98.8	1.0	0.7–1.3
Age (years)						
Median, range	36 (18–65)		41 (18–78)		0.96	0.95–0.98
Profession						
Teacher	125	1.0	12,887	99.0	Ref.	NA
Preschool educator	85	1.9	4,351	98.1	2.0	1.5–2.7
Reported contacts to SARS-CoV-2-positive individuals outside work^b^						
No	20	1.3	1,510	98.7	Ref.	NA
Yes	8	7.8	94	92.2	6.4	2.4–15.7
Reported contacts to SARS-CoV-2-positive individuals at work^b^						
No	23	1.8	1,290	98.2	Ref.	NA
Yes	5	1.6	316	98.4	0.9	0.3–2.4

CI: confidence interval; NA: not applicable; OR: unadjusted odds ratio; SARS-CoV-2: severe acute respiratory syndrome coronavirus 2.

^a^ Missing data for sex (n = 62).

^b^ Data from one testing site (A) only.

The best set of associated factors with SARS-CoV-2 infection involved staff age (p < 0.001), occupation (p < 0.001), and calendar week (p < 0.001). The bootstrap repetition resulted in a selection frequency of each of these variables as follows: age (100%), occupation (100%), calendar week (100%) (Supplementary Analysis S1: Data collected at Sites A and E).

## Discussion

Our SARS-CoV-2 screening programme in educational settings, which spanned the second half of 2020 when the COVID-19 pandemic progressed from a low level to an intense second wave, suggests elevated SARS-CoV-2 infection odds in preschool educators as compared with teachers. Notably, proportion of positive tests among all educational staff increased with rising incidence observed in the community, but not nearly to the same extent.

The increased proportion of positive SARS-CoV-2 tests in preschool educators may reflect routine physical contact to young preschool children, and thus increased potential viral exposure. This may also apply to younger school-age children, as reflected by similar proportion of positive tests in preschool educators and primary school teachers in a small subset of our study. Concurrently, physical distancing or facemask rules were less strictly set in Germany for preschool educators who work with children aged 0–5 years, as compared with teachers working with children and adolescents aged 6 years and older. Beyond occupational exposure, socioeconomic status and SARS-CoV-2 infection are inversely associated [11,12]. Becoming a preschool educator in Germany does not require university education and usually yields a below-average salary, which may indicate an average lower socioeconomic status compared to teachers. Moreover, preschool educators were rather young, and age was among the set of variables associated with infection. Presumably, this could reflect a more active social life at a younger age. By February 2022, educational staff in Germany in over 90% has received at least two doses of SARS-CoV-2 vaccination. This figure is in the range of two thirds and below 25% for children aged 12–17 and 5–12 years, respectively [13,14]. Increasing vaccination coverage among students may additionally reduce risk of infection in educational staff, which would require a renewed risk assessment for confirmation.

Data from the UK [15] and Italy [16] show a SARS-CoV-2 infection incidence in teachers that is comparable to other working adults of similar age. Concomitantly, data from Norway suggest no increased risk or even a lower risk for teachers of children of any age and childcare workers during the first pandemic wave, and a moderately increased risk of infection during the second wave; this may be explained by their higher testing activity compared with the general population [17]. In the present study, unweighted proportion of positive SARS-CoV-2 tests in teachers from all districts of Berlin was 1.2%, while in a simultaneous survey on the adult population of a central district, it was 0.9% [10]. In another study, conducted in early November 2020 at peak transmission in Berlin, 1.4% (2/140) teachers were found to be infected [5]. Together, this suggests that infection risk in teachers was not substantially increased.

The proportion of positive SARS-CoV-2 tests in educational staff did not increase to the extent community incidence did in the second pandemic wave, and was even observed to drop at a time when community incidence rose to its peak. Conceivably, this might reflect a protective impact of the educational job setting under IPC conditions, including strict home isolation for positive cases. An indirect proof of acceptably safe working conditions can be derived from the fact that contacts to SARS-CoV-2-positive individuals in the private – but not the occupational – context were most strongly associated with SARS-CoV-2 infection. Likewise, in autumn 2020, Austrian teachers were the origin of up to 90% of SARS-CoV-2 school clusters, contrasting their relative minority at school [18], supporting the notion of relative workplace safety. Similarly, an Italian study performed during the second wave (autumn 2020–spring 2021) showed that secondary infections of teachers were rare and more likely if the index case was a teacher than a student (37% vs 10%) [16]. This underlines the necessity of adjusting social behaviours to control overall viral transmission, but also of further occupational IPC measures. Infection risks stemming from the private context do not argue against routine SARS-CoV-2 testing in schools two to three times per week, as such will detect infections otherwise introduced into educational facilities. Increased proportion of positive tests in preschool educators also supports their prioritised vaccination.

### Limitations

Our study has several limitations. Of all participants, we estimate 15% who were repeatedly tested. Under-reporting of symptoms (presumed to lead to exclusion from screening) and over-reporting (misunderstood test indication) cannot be excluded. Among all participants, 5% reported symptoms at time of presentation. Selection bias because of easily accessible testing might apply. At two testing sites, we were able to identify and exclude staff other than teachers or preschool educators, e.g. kitchen staff, and it is possible that a similar proportion of ca 15% applies to the remaining dataset. Nevertheless, re-running the analysis with and without exclusion of additional staff did not yield different results, suggesting that our overall findings are not substantially affected. Unfortunately, we could not differentiate between primary and secondary school teachers for the whole study group. In the small respective subset, the proportions of positive tests between teachers of the two age groups did not differ significantly.

Comparing the proportion of positive tests to community incidence data has inherent limitations. Community incidence is based on notified symptomatic patients or primary contacts, the detection of which is subject to changing testing indications, access to testing, and test willingness. Translating the peak weekly incidence of over 200 per 100,000 in Berlin to a proportion of positive tests over 7 days of ca 0.2% provides a figure that is substantially lower than the simultaneous population prevalence of 0.9% [10]. Anti-SARS-CoV-2 antibody assessments from the general population reveal incidence data to be largely underestimated [19]. An actual comparison of our proportion of positive tests data with the simultaneous population prevalence data would have limitations, but also points to the mere absence of representative prevalence data in Germany. Absence of complete datasets for all participants might have impacted the detection and estimates of associated factors. These limitations need to be balanced against our large sample size.

## Conclusions and outlook

Preschool educators as compared with teachers had increased odds of SARS-CoV-2 infection during the second half of 2020 in Berlin. Private but not occupational contacts to SARS-CoV-2 infected individuals were found increase infection risks. At the beginning of 2022, vaccination coverage among educational staff was very high. In December 2021, vaccination coverage among adolescents (12–17 years) had reached more than 50%, and immunisations of children (aged 5–11 years) had started [13,20-22]. At the same time, the highly transmissible and possibly less virulent SARS-CoV-2 Omicron variant of concern (B.1.1.529 of the Phylogenetic Assignment of Named Global Outbreak (Pango) lineage designation), is causing very high infection activity, and the emergence of further variants cannot be excluded. In this ambiguous and volatile situation, gradually lifting IPC measures in the educational context requires continuous monitoring of the infection status to allow for prompt response in case of changing conditions.

## Data and code availability

The identified data are available upon reasonable request from the corresponding author.

Analysis code is available upon request from Welmoed van Loon, welmoed.van-loon@charite.de

## Supplementary Data

418000 Supplement
