# Supplementary material for: SARS-CoV-2 infection among educational staff in Berlin, Germany, June to December 2020
Source: Euro Surveill. 2022 Mar 17;27(11):2100524. doi: 10.2807/1560-7917.ES.2022.27.11.2100524 (PMC8971916; doi:10.2807/1560-7917.ES.2022.27.11.2100524)
Supplement: Supplement [file 21-00524_GERTLER_Supplement.pdf]

Supplementary Material

SARS-CoV-2 infection among educational staff in Berlin, Germany, June to December 2020

Supplemental methods for backwards stepwise selection procedure

"This supplementary material is hosted by Eurosurveillance as supporting information alongside the article SARS-CoV-2 screening in educational staff in Berlin, Germany, June to December 2020, on behalf of the authors, who remain responsible for the accuracy and appropriateness of the content. The same standards for ethics, copyright, attributions and permissions as for the article apply. Supplements are not edited by Eurosurveillance and the journal is not responsible for the maintenance of any links or email addresses provided therein."

We explored which combination of variables in our dataset best described the SARS-CoV-2 infection in a logistic regression applying backward stepwise selection by Akaike information criterion (AIC). The following variables were included for this process: SARS-CoV-2 infection status (positive/negative), sex (male/female), age (years), testing site (A/B/C/D/E) and time (by calendar week).

We fitted a binomial logistic regression model including all variables above, with SARS-CoV-2 infection as outcome variable. Then we used this model for backwards selection by AIC (R function *stepAIC* from the *MASS* package in R). For the bootstrap analysis, this process was repeated on 1,000 resampled datasets (R function *boot.stepAIC* from the *bootStepAIC* package in R) as follows: a new dataset was simulated by resampling with replacement; the full binomial logistic regression model was fitted; the backwards stepwise selection by AIC was done on the new full model; among the 1,000 resulting selected models, the number of times was counted that each variable was selected.

Supplementary Table S1 Distribution of test population at each test site

	Testing site									
	**A N=2287**		**B N=4542**		**C N=2908**		**D N=4427**		**E N=3284**	
	%	n	%	n	%	n	%	n	%	n
Positive SARS-CoV-2 PCR result	1.3	30	1.0	45	1.7	49	1.1	50	1.1	36
Profession										
Teacher	47.9	1095	82.0	3724	78.1	2271	77.6	3436	75.7	2486
Preschool eduator	52.1	1192	18.0	818	21.9	637	22.4	991	24.3	798
Age, median (range)	38 (18-73)		42 (18-73)		40 (18-77)		41 (18-70)		42 (18-78)	
Female	74.2	1698	73.9	3352	71.0	2062	72.1	3188	73.8	2386
Reported symptoms	10.5	239	NA		NA		NA		1.9	62

Supplementary analysis S1: Data collected at site A and E *with* and *without* exclusion of non-teacher and non-preschool educator participants.

One of the constraints in our full dataset was the lack of more detailed information on the function that staff in educational facilities fulfill for most participants. At two testing sites, educational staff not being teacher or preschool educator could be identified and excluded. However, it is possible that a similar proportion of some 15% applies to the remaining data set. To investigate the impact of this "noise" in the occupation variable, we repeated the analyses for effect estimation of occupation and contact history on SARS-CoV-2 infection status in two data subsets: data collected at site A and E *with* and *without* exclusion of non-teacher and non-preschool educator participants.

Excluded participants did not differ from the included participants as for SARS-CoV-2 infections status, sex, age, reported symptoms, and contact history. However, the excluded participants were more frequently categorized as preschool educator than teacher (Supplementary Table S2) was. The outcomes of our models were almost similar with and without exclusion of non-teachers and non-preschool educators (Supplementary Figure S1).

Supplementary Table S2. Characteristics of excluded participants and included participants from testing site A and E. Participants were excluded because they could be identified to not be a teacher or preschool educator, *e.g.*, facilitating staff in educational facilities.

	Excluded participants (n=987)		Included participants (n=5584)		OR	95% CI
	n	%	n	%		
**Result of SARS-CoV-2 PCR**						
Negative	972	15.1	5484	84.9	Ref.	NA
Positive	14	17.5	66	82.5	0.8	0.5-1.6
**Profession before inspection of additional data**						
Teacher	380	9.6	3583	90.4	Ref.	NA
Preschool educator	607	23.3	2001	76.7	0.4	0.3-0.4
**Age (median, range)**	39 (18-73)		41 (18-78)		NA	NA
**Sex**						
Male	281	16.4	1437	83.6	Ref.	NA
Female	706	14.7	4096	85.3	1.1	1.0-1.3
**Any symptoms**						
No	936	15.1	5250	84.9	Ref.	NA
Yes	51	14.4	302	85.6	1.1	0.8-1.5
**Reported contacts to SARS-CoV-2-positive individuals outside work [a]**						
No	596	28.0	1531	72.0	Ref.	NA
Yes	28	21.5	102	78.5	1.4	0.9-2.3
**Reported contacts to SARS-CoV-2-positive individuals at work [a]**						
No	528	28.7	1314	71.3	Ref.	NA
Yes	96	23.0	321	77.0	1.3	1.0-1.7

CI: confidence interval; NA: not applicable; OR: unadjusted OR.

[a] Data from one testing site A) only.

Supplementary Figure S1. Comparison of effect estimates for SARS-CoV-2 infection in a dataset *with* and *without* exclusion of non-teachers and non-preschool educators e.g., facilitating staff in educational settings). Effect estimates for occupation teacher/preschool educators) were obtained by a logistic regression including sex, age and calendar week as covariates. For the variables on contact history, the covariate set included occupation, sex, age, and calendar week.

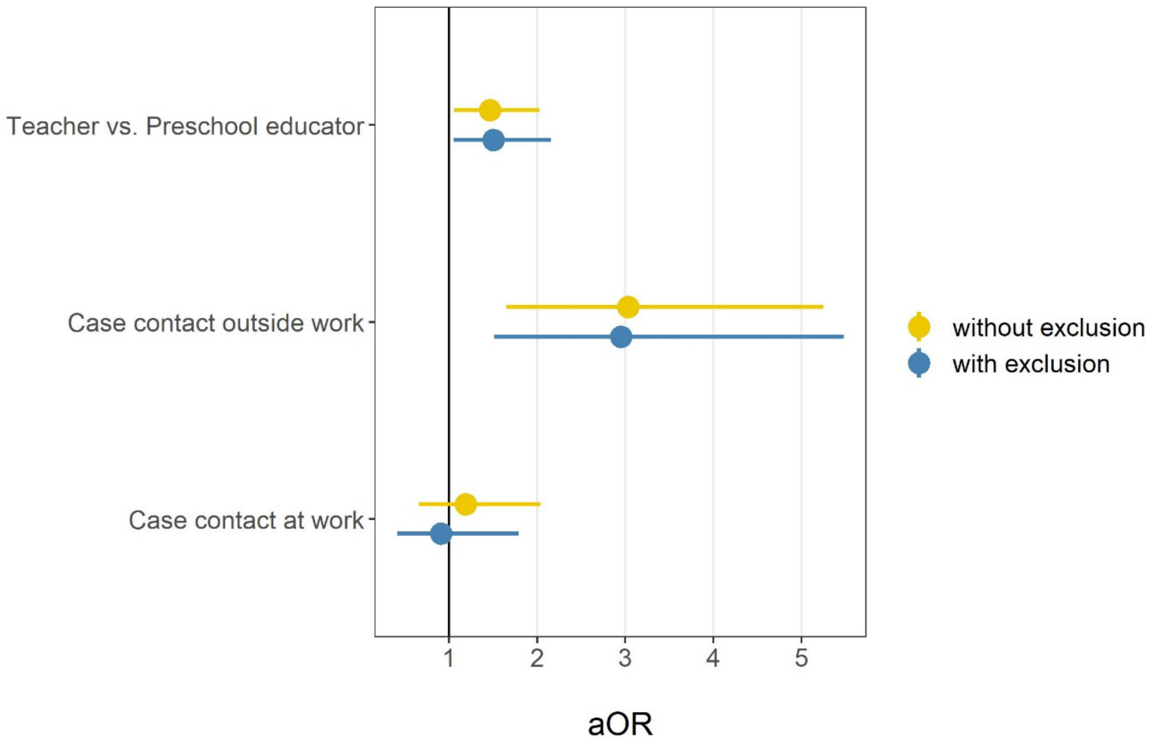

Supplementary Table S3. Questionnaire for the Screening of special occupational/population groups. Systematically used at testing site A and partially at testing site E.

1. Where do you work?
    a. Elementary school
    b. Secondary school/gymnasium
    c. Kindergarten
    d. Nursing or geriatric care, in-patient homes, nursing communes)
    e. Nursing or geriatric care, outpatient
    f. Other community facility
    g. Correctional facility
    h. Cultural institution
    i. Passenger transport
    j. Gastronomy Other facility
2. Do you mainly or exclusively core activity) work directly with "clients" or the target group of your facility? Depending on the type of facility, e.g. schoolchildren, kindergarten children, nursing cases, prisoners, driving) guests, etc.
    a. Yes mainly direct contact with the target group)
    b. No mainly in activities remote from the target group, e.g. management, administration, etc.)

    2.1. If 2. was answered with "yes":

    How many different people e.g., students, daycare children, care cases, prisoners, guests) do you teach/care for/provide/transport/serve on average per week? based on the last 14 days)

    a. < 8 people
    b. 9 - 16 children/youth/persons
    c. 17 - 24 children/youth/persons
    d. 25 - 32 children/youths/persons
    e. 32 - 40 children/youth/persons
    f. 41 - 60 persons
    g. 61 - 100 persons
    h. > 100 persons
3. How many hours per week did you last work at the facility?
    a. 10 hours per week
    b. 10 - 19.5 hours per week <20 hours)
    c. 20 - 30 hours per week
    d. 31 - 40 hours per week
    e. > 40 hours
4. What specific infection control measures are currently within the last 14 days) in place at your workplace for staff?
    a. Wearing of a mouth-nose-covering
    b. Regular airing
    c. Regular hand washing/disinfection
    d. Distance rule min. 1.5m distance)

5. What specific infection control measures are currently in place within the last 14 days) at your workplace for members of the facility students, daycare center children, residents...)?
    a. Wearing of a mouth-nose-mask
    b. Regular airing
    c. Regular hand washing/disinfection
    d. Distance rule min. 1.5m distance)
6. Are mouth-to-nose masks whether medical or those without special filters) provided at your facility?
    a. Yes
    b. No
7. Is hand sanitizer provided at your facility?
    a. Yes
    b. No
8. Have you generally been instructed by your employer to call in sick and stay away from the facility if you have symptoms of illness?
    a. Yes
    b. No
9. What is your current living situation?
    a. alone
    b. Together with family/shared apartment
10. Have you traveled within the past 14 days?
    a. Yes, abroad
    b. Yes, within Germany
    c. No
11. Only for trips abroad - select the country/countries of travel

    Drop-down menus with all countries for at least 2 foreign trips

12. Have you had any acute symptoms of illness fever, cough, sore throat, headache) within the last 7 days?
    a. Yes
    b. No
13. Have you noticed any loss of smell or taste within the last 7 days?
    a. Yes
    b. No
14. Have you had contact with a confirmed Covid-19 case in the last 14 days?
    a. Yes, in private environment
    b. Yes, at work
    c. No
15. Have you been tested for SARS-CoV-2 swab) in the past?
    a. No
    b. Yes, 1x
    c. Yes, 2x
    d. Yes, 3x or more
16. Have you had Covid-19 in the past and/or tested positive once?
    a. No
    b. Yes

17. Do you suffer from any chronic diseases)?
    a. Heart disease
    b. Lung disease
    c. Immunodeficiency
    d. Diabetes diabetes)
    e. Overweight alternatively extra question about height and weight)
    f. Other chronic diseases

Supplementary Table S4. Questionnaire for the Screening of special occupational/population groups. Systematically used at testing site E.

1. How old are you?
    a. <40
    b. 40-50
    c. 51-60
    d. 61-70
    e. 71-80
    f. >80"
2. What is your current living situation?
    a. living alone
    b. family/ shared apartment/ institution
3. Do you privately care for or support one or more persons with age-related ailments, chronic illnesses or frailty at least once a week?
    a. Yes
    b. No
4. Do you work in any of the following areas?
    a. medical field
    b. school/ day care/ etc.
    c. No, in none
5. Do you smoke?
    a. Yes
    b. No
6. Are you pregnant?
    a. Yes
    b. No
    c. Don't know
7. Have you had close contact with a confirmed case?
    a. Yes
    b. No

    7.1. If 7. Was answered with yes:
        Date of Contact YYYYMMDD

8. Have you had a fever over 38°C) in the last 24 hrs?
    a. Yes
    b. No

9. Have you had a fever over 38°C) in the last 4 days?
    a. Yes
    b. No

8/9.1. If 8. or 9. Was answered with yes:

What was the highest temperature approx.?

a. 38
b. 39
c. 40
d. 41
e. 42
f. over 42
g. don't know

10. Which of the following symptoms have you had in the last 24 hrs?
    a. Chills
    b. no chills
11. Which of the following symptoms have you had in the last 24 hrs?
    a. aching limbs
    b. no pain in the limbs
12. Which of the following symptoms have you had in the last 24 hrs?
    a. loss of taste or smell
    b. no loss of taste or smell
13. Which of the following symptoms have you had in the last 24 hrs?
    a. listless and fatigued
    b. not tired and fatigued
14. Which of the following symptoms have you had in the last 24 hrs?
    a. persistent cough
    b. no persistent cough
15. Which of the following symptoms have you had in the last 24 hrs?
    a. Cold
    b. no cold
16. Which of the following symptoms have you had in the last 24 hrs?
    a. Diarrhoea
    b. no diarrhoea
17. Which of the following symptoms have you had in the last 24 hrs?
    a. sore throat
    b. no sore throat
18. Which of the following symptoms have you had in the last 24 hrs?
    a. Headache
    b. no headache
19. In the last 24 hrs, have you been out of breath more quickly than usual?
    a. Yes
    b. No

10-19.1. If at least one out of 10. – 19. Was answered with yes:

Date of symptoms YYYYMMDD

20. Have you been diagnosed with chronic lung disease by a doctor?
    a. Yes
    b. No
    c. Don't know
21. Have you been diagnosed with diabetes by a doctor?
    a. Yes
    b. No
    c. Don't know
22. Have you been diagnosed with heart disease by a doctor?
    a. Yes
    b. No
    c. Don't know
23. Have you been diagnosed with obesity by a doctor?
    a. Yes
    b. No
    c. Don't know
24. Are you currently taking cortisone in tablet form?
    a. Yes
    b. No
    c. Don't know
25. Are you currently taking immunosuppressants?
    a. Yes
    b. No
    c. Don't know
26. Have you had a flu vaccination in the period from 1 August 2020 to the present?
    a. Yes
    b. No
